# Predicting Delayed Extubation After General Anesthesia in Postanesthesia Care Unit Patients Using Machine Learning: Model Development Study

**DOI:** 10.2196/72602

**Published:** 2025-11-11

**Authors:** Jianwei Luo, Shaoman Lin, Liman Wang, Huanfan Ji, Jingcong Zheng, Tingkang Wang, Lin Chen, Ziqi Lin, Zhongqi Liu, Ning Liufu

**Affiliations:** 1 Department of Anesthesiology, Sun Yat-sen Memorial Hospital, Sun Yat-sen University, 107 Yanjiang West Road, Guangzhou, 510120, China, 86 020-81332199; 2 Department of Anesthesiology, Shenshan Medical Center, Memorial Hospital of Sun Yat-sen University, Shanwei, China

**Keywords:** delayed extubation, general anesthesia, postanesthesia care unit, machine learning algorithms, XGBoost, extreme gradient boosting

## Abstract

**Background:**

Delayed extubation after general anesthesia increases complications and can lead to longer hospital stays and higher mortality. Current risk assessments often rely on subjective judgment or simple tools, whereas machine learning offers potential for real-time evaluation, though research is limited and typically uses single-algorithm models.

**Objective:**

The aims of this study were to identify risk factors for delayed extubation after general anesthesia in the sample and to construct a risk prediction model for delayed extubation in this population.

**Methods:**

Data from 4779 patients admitted to the postanesthesia care unit between September 2023 and May 2024 were used to develop prediction models for delayed extubation using k-nearest neighbor, decision tree, extreme gradient boosting, random forest, a light gradient boosting machine, and an artificial neural network. Model performance was assessed by calculating the area under the receiver operating characteristic curve, sensitivity, specificity, accuracy, *F*_1_-score, and Brier score. Calibration performance was evaluated using calibration curves generated with 100-bin quantile calibration and Loess smoothing to provide bias-corrected and smoothed visual assessment. Additionally, the Hosmer-Lemeshow goodness-of-fit test was performed to quantitatively evaluate calibration, with *P* values >.05 indicating good calibration.

**Results:**

Among the 6 models evaluated, the extreme gradient boosting model demonstrated the best performance, with an area under the receiver operating characteristic curve of 0.750 (95% CI 0.703‐0.796), a sensitivity of 0.734 (95% CI 0.635‐0.827), and a specificity of 0.647 (95% CI 0.623‐0.673). The model calibration was acceptable, with a Brier score of 0.0505 and a nonsignificant Hosmer-Lemeshow goodness-of-fit test (χ²_6_=7.3; *P*=.287), indicating good calibration. Shapley additive explanations were used to rank feature importance.

**Conclusions:**

These machine learning models enable early identification of delayed extubation risk, supporting personalized clinical decisions and optimizing postanesthesia care unit resource allocation.

## Introduction

### Background

Extubation is a critical step in the recovery process following general anesthesia. More than 20% of major airway management–related complications occur immediately before or after extubation, with severe consequences such as hypoxia and mortality [1,2]. Prolonged intubation offers no benefit; delayed extubation may adversely affect the patient’s cardiovascular and respiratory systems, resulting in complications such as ventilator-associated pneumonia, pulmonary fibrosis, ventilator dependency, and arrhythmias [3]. Delayed extubation after general anesthesia not only reduces the efficiency of the transition from the postanesthesia care unit (PACU) to the ward but also slows functional recovery, exacerbates physiological and psychological stress responses, and diminishes the quality of the patient’s resuscitation period. This increases the mental and economic burden on the patient and family and raises the incidence of medical disputes [4].

Screening and predicting patients with a high risk for delayed extubation is crucial for optimizing PACU operations and patient care. Several demographic features have been identified as significant predictors, including advanced age [5,6] and the presence of comorbidities such as chronic obstructive pulmonary disease (COPD), cardiovascular diseases, and diabetes [7]. Additionally, a high BMI has been linked to increased respiratory complications, potentially prolonging the need for ventilatory support [8,9]. Furthermore, the nature of the surgical procedure itself significantly influences the likelihood of delayed extubation. Complex and prolonged surgeries, particularly those involving the thoracic or cardiovascular systems, can lead to increased physiologic stress and a higher risk of postoperative complications that may necessitate extended intubation [10]. Significant hemorrhage can result in hemodynamic instability, which can affect the patient’s ability to maintain adequate oxygenation and ventilation postoperatively [11-13]. In clinical practice, extubation following general anesthesia is predominantly executed by medical and nursing professionals within the PACU. However, current research provides limited information on the impact of nursing-related monitoring and management variables within the PACU on the extubation time.

There are increasing studies on building machine learning predictive models for delayed extubation. Previous studies have suggested that machine learning algorithms, including extreme gradient boosting (XGBoost), gradient boosting machine, and artificial neural networks, have advantages in constructing predictive models for delayed extubation in the intensive care unit [14,15]. However, for postoperative delayed extubation, most studies used logistic regression for developing a nomogram model or a single machine learning algorithm for constructing a predictive model [9]. Fewer studies use multiple machine learning algorithms to build a postoperative delayed extubation prediction model following general anesthesia in the PACU.

Therefore, this study aimed to explore perioperative features, including monitoring and management variables in the PACU, associated with delayed extubation after general anesthesia and to develop prediction models for delayed extubation using machine learning algorithms. It is hypothesized that machine learning models incorporating PACU monitoring and management variables can be developed to predict delayed extubation, enabling early assessment and prevention of this adverse event following general anesthesia.

Delayed extubation after general anesthesia is a common postoperative complication associated with increased adverse events, such as prolonged hospital stays, higher intensive care needs, and elevated mortality rates. Identifying patients with high risk and optimizing recovery care is crucial. Current risk assessments often rely on subjective judgment or simplistic tools, which may overlook the complex patient profiles and intraoperative dynamics. Machine learning, with its robust data mining and predictive capabilities, offers real-time evaluation and intervention potential. However, research specifically addressing delayed extubation remains limited, with most studies relying on traditional single-algorithm models.

### Aims

The aims of this study were to identify the risk factors for delayed extubation after general anesthesia in the sample and to construct a risk prediction model for delayed extubation in this population.

## Methods

### Machine Learning Model Selection

The data extracted from electronic medical records in this retrospective cohort study can be used to substantially predict the risk of delayed extubation after general anesthesia. In line with our research objectives, we selected 6 predictive modeling methods for analysis: random forest (RF), XGBoost, k-nearest neighbor (KNN), decision tree, light gradient boosting machine (LightGBM), and artificial neural networks.

### Data Collection

Patient consent was waived due to the retrospective nature of the study. Patients transferred to the PACU after general anesthesia at Shenshan Medical Center in Shanwei, China, from September 2023 to May 2024 were retrospectively enrolled.

### Variables

Demographic information included sex, age, American Society of Anesthesiologists (ASA) classification, BMI, smoke history, and comorbidities (including cerebral infarction and COPD).

Preoperative examinations included red blood cell distribution width; alanine and aspartate aminotransferase levels; serum sodium concentration, serum concentrations of sodium, potassium, calcium, hemoglobin, and creatinine; and chest x-ray findings. Preoperative examinations were performed within 7 days preoperatively, and the latest result was included in case the same test was conducted multiple times.

Surgery-related parameters included intraoperative infusion volume, intraoperative blood loss, duration of surgery, case condition (usual, urgent, difficult, and critical), site, and level of surgery. Intraoperative infusions were determined by the anesthesiologist based on the patient’s intraoperative vital signs, urine output, or hemodynamic monitoring.

PACU monitoring and management indicators included tympanic temperature following PACU admission, time to extubation in PACU, and the sufentanil administered before extubation in the PACU. The supplemental dose of sufentanil was administered at 0.1 µg/kg per dose, with adjustments made based on the patient’s specific resuscitation process and the severity of postoperative pain.

### Study Population and Data Extraction

#### Eligibility Criteria

The inclusion criteria were patients who underwent tracheal intubation under general anesthesia and were transferred to the PACU postoperatively. The exclusion criteria were as follows: (1) extubation before admission to the anesthesia recovery room, (2) preoperative coma, (3) presence of severe mental illness or psychiatric disorders, and (4) missing data exceeding 30% of the required information. Patients’ demographic information, comorbidities, preoperative examinations, surgery-related parameters, and PACU monitoring and management indicators were retrieved from the institutional database.

#### Anesthesia Method

No premedication was used for any patients. Induction was administered by intravenous injection of sufentanil (0.3‐0.5 μg/kg), propofol (1.5‐2.5 mg/kg), and rocuronium (0.6 mg/kg). Alternatively, mivacurium (0.07‐0.25 mg/kg) or cisatracurium (0.2 mg/kg) was used for muscle relaxation. After achieving the appropriate anesthesia depth and muscle relaxation, intubation was performed for mechanical ventilation (Tidal Volume 6‐8 ml/kg, Respiratory Rate 10‐12 breaths/min). Intraoperative parameters were managed, and patients were transferred to the PACU within 5 minutes after surgery.

### Data Analysis

Statistical analyses were performed using Python (version 3.12.4; Python Software Foundation). Variables with ≥30% missing data were excluded from the analysis, while those with <30% missing data were imputed using the KNN classification algorithm. Continuous variables were assessed for normality and compared using the independent 2-tailed *t* test or the Mann-Whitney *U* test, as appropriate. Categorical variables were compared using the chi-square test or the Fisher exact test, depending on expected cell counts. All statistical tests were 2-sided, and a *P* value <.05 was considered statistically significant.

Feature selection was conducted on the training set using the least absolute shrinkage and selection operator (LASSO) to identify the most predictive variables. During the evaluation phase, a threshold optimization strategy was applied to determine clinically meaningful cut-off points. Specifically, thresholds were selected under dual constraints, requiring both sensitivity and specificity to be ≥0.60, to ensure that the model achieved a balance between correctly identifying positive cases and minimizing false positives. When no threshold met these dual criteria, the Youden index was used as an alternative to identify the optimal threshold. This approach ensured that the final model outputs had practical applicability for clinical risk stratification.

Model discrimination performance was evaluated in the test set by calculating the area under the receiver operating characteristic curve (AUROC), alongside sensitivity, specificity, positive predictive value (PPV), and negative predictive value (NPV). CIs for these metrics were estimated using 1000 bootstrap resampling iterations to enhance the robustness of performance assessment.

Calibration performance was evaluated using calibration curves generated with 100-bin quantile calibration and LOESS smoothing; in the LOESS procedure, the parameter frac=0.3 indicates that 30% of the data were used for each local fit to control the degree of smoothing. The Hosmer-Lemeshow goodness-of-fit test was also performed to quantitatively assess calibration, with *P* values >.05 indicating good calibration.

### Data Processing and Feature Selection

Delayed extubation after general anesthesia has been defined as the removal of the tracheal tube 60 minutes or more after general anesthesia [16]. This study defined delayed extubation as removal of the tracheal tube over the same time frame following PACU admission, while removal in less than 60 minutes was categorized as nondelayed extubation.

Continuous variables were presented as mean with SD or median with IQR according to normality for the variables. Categorical variables were expressed as frequencies with percentages. Missing values were filled in using the KNN classification algorithm, which estimates missing values based on the values of patients with similar features. Patients ultimately enrolled in this study were randomized in a 7:3 ratio into primary and test cohorts. We used the optimal thresholds from receiver operating characteristic (ROC) curve analysis to convert continuous variables, such as intraoperative infusion volume and duration of surgery from the training set, into categorical variables [17].

The LASSO was used to identify key features associated with delayed extubation after general anesthesia for the construction of the machine learning models. The machine learning algorithms used in this study included KNN, decision tree, XGBoost, RF, LightGBM, and artificial neural networks. To enhance model performance, parameter tuning was conducted on the training set using a 10-fold cross-validation approach.

### Model Evaluation

The AUROC, sensitivity, specificity, PPV, NPV, accuracy, and *F*_1_-score were chosen as primary metrics for model evaluation. To enhance the robustness of performance assessment, 1000 bootstrap resampling iterations were performed to estimate the CIs for these metrics. Model calibration was evaluated using calibration curves generated with 100-bin quantile calibration and Loess smoothing (frac=0.3) to provide bias-corrected and smoothed visual assessment, and the Hosmer-Lemeshow goodness-of-fit test was performed to quantitatively assess calibration, with *P* values >.05 indicating good calibration. Additionally, Brier scores were calculated to assess overall calibration accuracy.

### Ethical Considerations

This study was approved by the institutional review board of Shenshan Medical Center, Memorial Hospital of Sun Yat-sen University (2024-SSKY-113-01). The trial was registered in the Chinese Clinical Trial Registry (ChiCTR2400090247). Because this was a retrospective study using deidentified clinical data and posed minimal risk to participants, the institutional review board waived the requirement for informed consent. Participant privacy and data confidentiality were strictly protected; all data were anonymized before analysis and used only for research purposes. No financial compensation was provided to participants.

## Results

**Figure 1.**
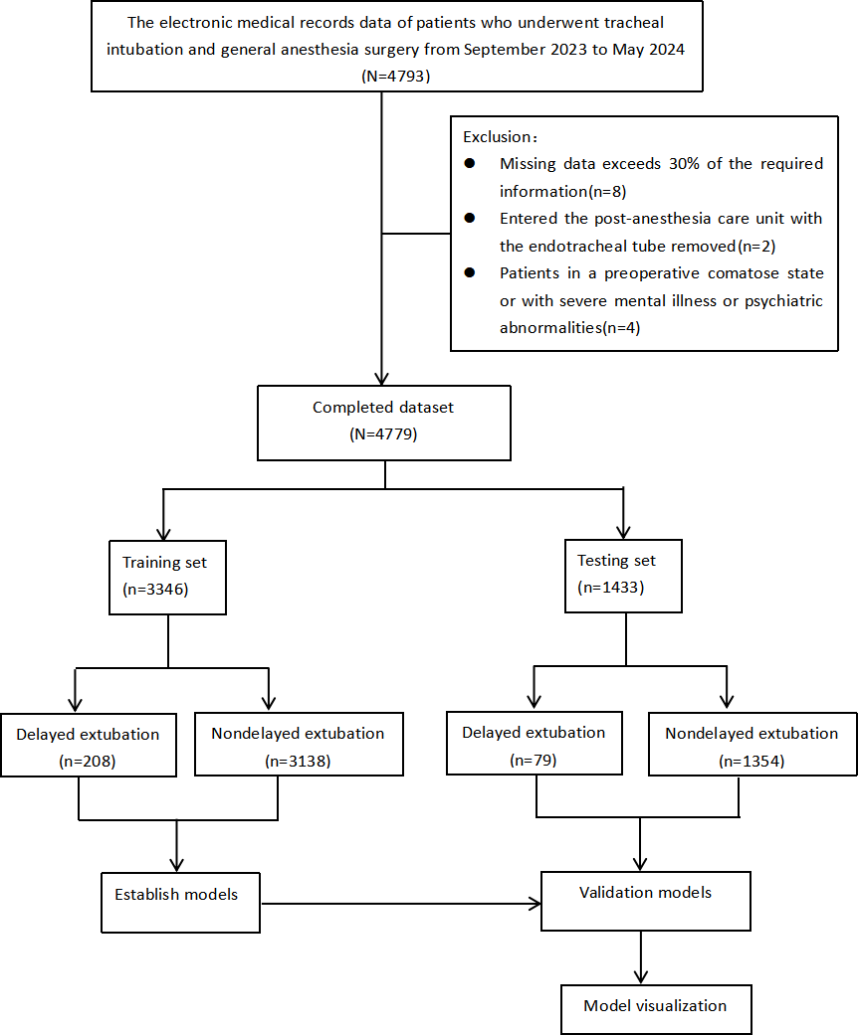
Patient recruitment flowchart

A total of 4793 patients were initially included in the study. On the basis of the exclusion criteria, 8 (0.16%) patients were excluded due to having more than 30% missing data, 2 (0.04%) patients were excluded because their tracheal tube had been removed before admission to the PACU, and 4 (0.08%) patients were excluded due to preoperative unconsciousness or severe psychiatric disorders. Therefore, 4779 patients were finally enrolled in the analysis, of whom 6% (287/4793) experienced delayed extubation, defined as extubation time of 60 minutes or more after admission to the PACU, and 93.72% (4492/4793) of patients did not experience delayed extubation. The dataset was randomly divided into primary and test cohorts in a 7:3 ratio, resulting in 70.01% (3346/4779) of patients being placed in the primary cohort and 29.99% (1433/4779) being placed in the test cohort. In the primary cohort, 6.21% (208/3346) of patients were assigned to the delayed extubation group and 93.78% (3138/3346) of patients were assigned to the nondelayed extubation group. The patient recruitment flowchart is shown in Figure 1.

**Figure 2.**
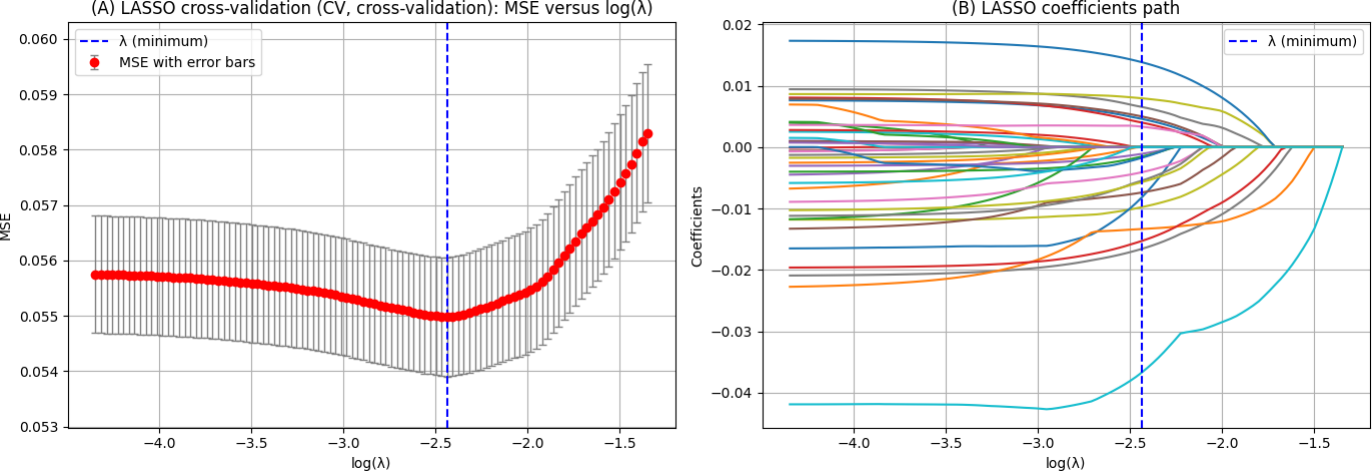
(A) Least absolute shrinkage and selection operator (LASSO) cross-validation curves and (B) coefficient path plot for LASSO regression analysis. MSE: mean square error.

No statistically significant differences were found in the univariate comparisons between the primary and test cohorts (Multimedia Appendix 1). The optimal parameter (λ) in the LASSO model was selected using 10-fold cross-validation. Dashed lines were drawn at the optimal value using the minimum criterion. A vertical line was drawn at the value chosen by 10-fold cross-validation, where the optimal λ resulted in 13 nonzero coefficient features (Figure 2). LASSO regression generated 13 nonzero coefficient variables at the value of λ (minimum)=0.0037 for building a machine learning prediction model, including age, sex, BMI, ASA classification, cerebral stroke history, intraoperative infusion volume, duration of surgery, history of COPD, case condition, surgical site, surgical level, tympanic temperature following PACU admission, and sufentanil administered before extubation in the PACU.

**Figure 3.**
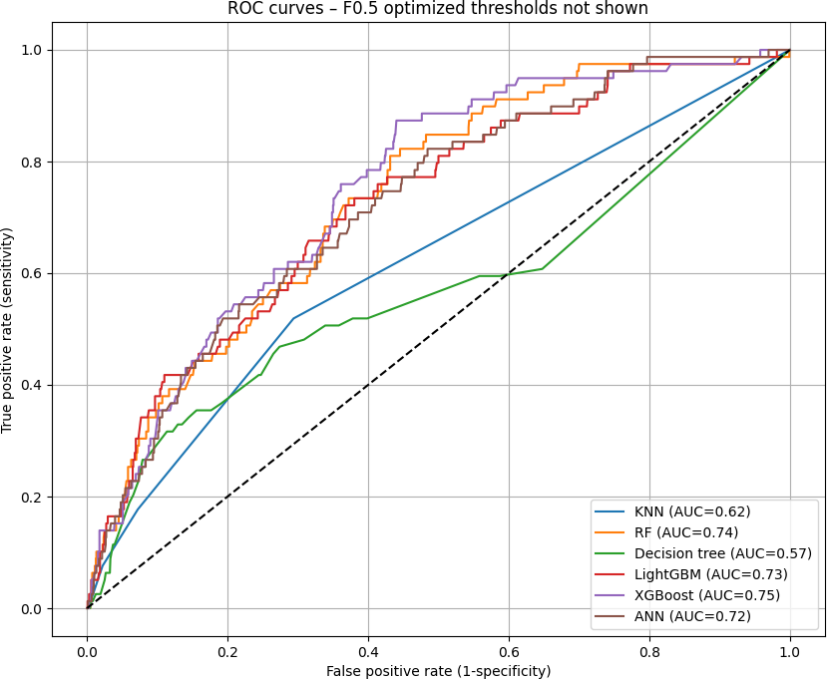
Receiver operating characteristic (ROC) curves for numerous machine learning delayed extubation prediction models. ANN: artificial neural network; AUC: area under the curve; KNN: k-nearest neighbor; LightGBM: light gradient boosting machine; RF: random forest; XGBoost: extreme gradient boosting.

On the basis of the test cohort, ROC (Figure 3) was drawn for different machine learning prediction models (KNN, decision tree, XGBoost, RF, LightGBM, and artificial neural networks). The XGBoost model demonstrated the highest overall performance among all models tested. In the test cohort, the XGBoost model achieved an AUROC of 0.750 (95% CI 0.703‐0.796), a sensitivity of 0.734 (95% CI 0.634‐0.827), a specificity of 0.647 (95%CI 0.622‐0.673), a PPV of 0.108 (95% CI 0.083‐0.134), a NPV of 0.976 (95%CI 0.966‐0.985; Table 1), an *F*_1_-score of 0.188, and accuracy of 0.652 (Table 2). The Brier score was 0.0505, indicating a low mean squared difference between predicted probabilities and actual outcomes and suggesting good model calibration (Table 2).

**Table 1.** Comparative performance of candidate models on the hold-out test dataset.

Model	AUROC^a^ (95%CI)	Sensitivity (95%CI)	Specificity (95%CI)	PPV^b^ (95%CI)	NPV^c^ (95%CI)
RF^d^	0.737 (0.684‐0.787)	0.696 (0.5976‐0.7945)	0.648 (0.6224‐0.6738)	0.103 (0.0790‐0.1295)	0.9734 (0.9628‐0.9836)
DT^e^	0.567 (0.493‐0.643)	0.316 (0.2208‐0.4211)	0.886 (0.8686‐0.9022)	0.139 (0.0913‐0.1894)	0.9569 (0.9455‐0.9679)
KNN^f^	0.620 (0.409‐0.630)	0.519 (0.4096‐0.6302)	0.706 (0.6825‐0.7311)	0.093 (0.0668‐0.1212)	0.9618 (0.9504‐0.9732)
LightGBM^g^	0.725 (0.671‐0.779)	0.734 (0.6375‐0.8261)	0.614 (0.5876‐0.6397)	0.100 (0.0769‐0.1254)	0.9754 (0.9651‐0.9851)
XGBoost^h^	0.750 (0.703‐0.796)	0.734 (0.6349‐0.8272)	0.647 (0.6227‐0.6733)	0.108 (0.0833‐0.1349)	0.9766 (0.9666‐0.9857)
Artificial neural network	0.719 (0.661‐0.775)	0.632 (0.5316‐0.7381)	0.672 (0.6468‐0.6966)	0.101 (0.0763‐0.1283)	0.9691 (0.9581‐0.9799)

a AUROC: area under the receiver operating characteristic curve.

b PPV: positive predictive value.

c NPV: negative predictive value.

d RF: random forest.

e DT: decision tree.

f KNN: k-nearest neighbor.

g LightGBM: light gradient boosting machine.

h XGBoost: extreme gradient boosting.

**Table 2.** Performance metrics, calibration assessment, and goodness-of-fit statistics for each machine learning model predicting delayed extubation.

Model	Accuracy	*F*_1_-score	Brier score	Hosmer-Lemeshow *Χ*^2^ (*df*)	*P* value
KNN^a^	0.695	0.158	0.0576	31.53 (6)	<.001
RF^b^	0.651	0.180	0.0516	17.16 (6)	.009
DT^c^	0.854	0.193	0.0629	2097.51 (6)	<.001
LightGBM^d^	0.621	0.176	0.0515	23.87 (6)	.001
XGBoost^e^	0.652	0.188	0.0505	7.38 (6)	.29
ANN^f^	0.669	0.174	0.0503	4.27 (6)	.64

a KNN: k-nearest neighbor.

b RF: random forest.

c DT: decision tree.

d LightGBM: light gradient boosting machine.

e XGBoost: extreme gradient boosting.

f ANN: artificial neural network.

**Figure 4.**
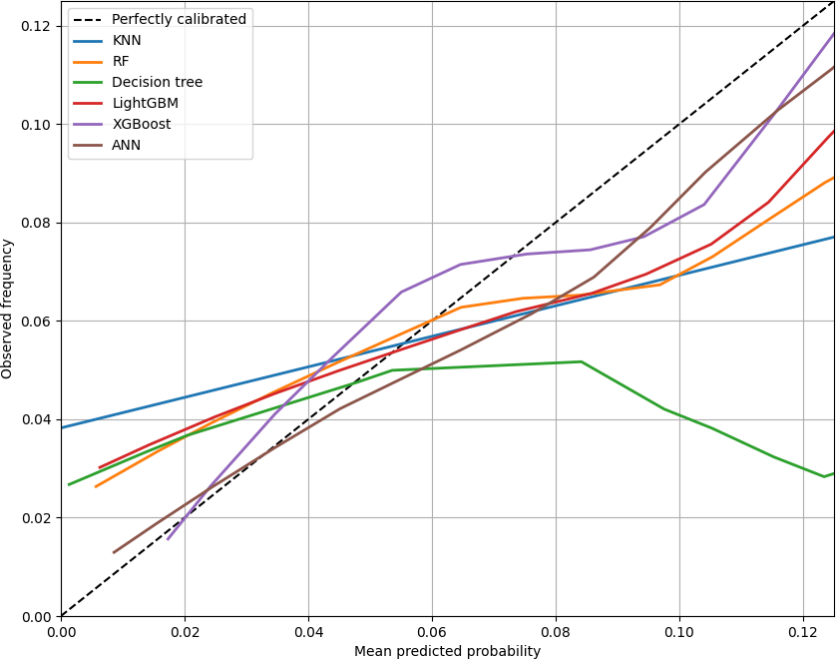
Calibration curve. ANN: artificial neural network; KNN: k-nearest neighbor; LightGBM: light gradient boosting machine; RF: random forest; XGBoost: extreme gradient boosting.

The calibration curve (Figure 4) showed good agreement between predicted probabilities and observed outcomes for the XGBoost model, with a Hosmer-Lemeshow goodness-of-fit test *χ*²_6_=7.38 and *P*=.287 (Table 2), indicating no significant lack of fit. These results suggest that the XGBoost model may serve as a reliable tool for predicting the risk of delayed extubation following general anesthesia in the PACU.

Similar trends were observed in the other models tested, although their overall discrimination performance and calibration were lower compared to XGBoost (Table 1 and Figure 3).

**Figure 5.**
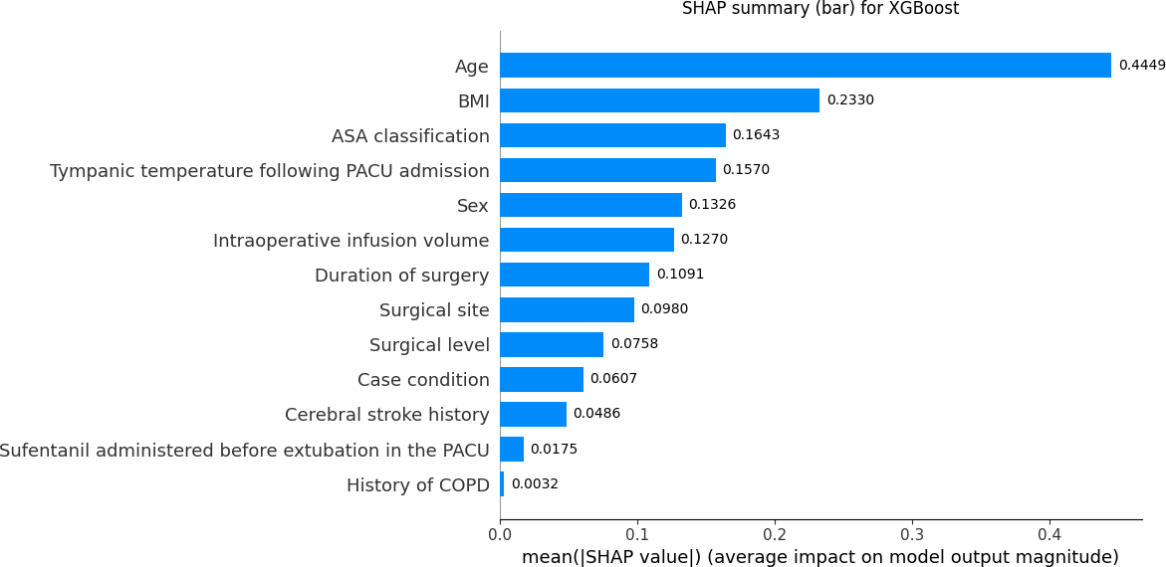
Bar chart of Shapley additive explanations (SHAP) for important features in the extreme gradient boosting (XGBoost) model. ASA: American Society of Anesthesiologists; COPD: chronic obstructive pulmonary disease; PACU: postanesthesia care unit.

**Figure 6.**
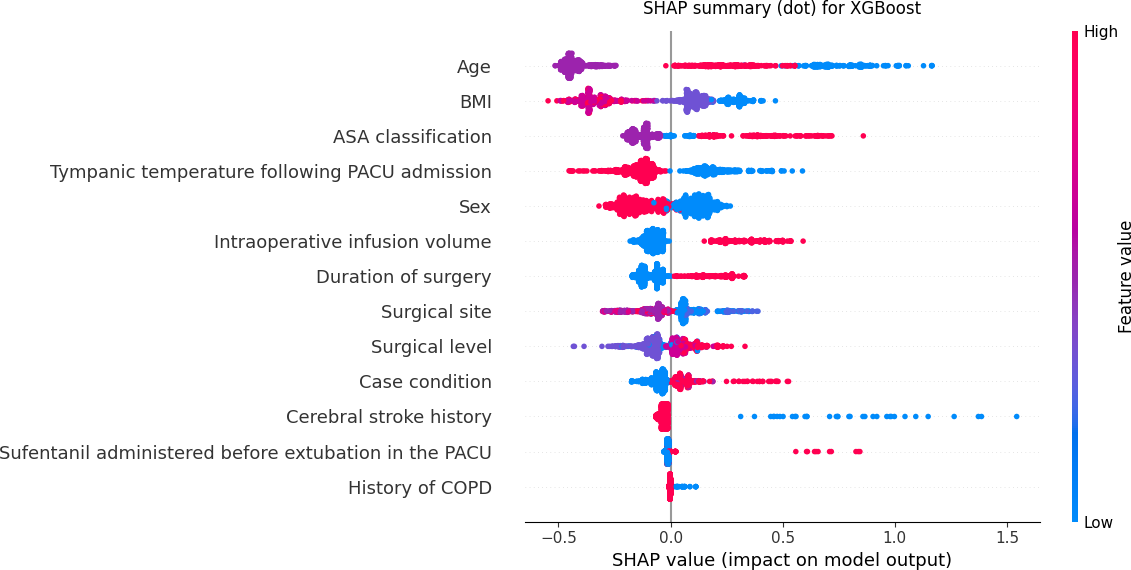
Global Shapley additive explanations (SHAP) summary for the extreme gradient boosting (XGBoost) model. Positive SHAP values increase the predicted probability of delayed extubation. Point color encodes the numeric feature code listed in Multimedia Appendix 2. Risk directions should be interpreted from SHAP values together with the coding scheme. ASA: American Society of Anesthesiologists; COPD: chronic obstructive pulmonary disease; PACU: postanesthesia care unit.

Given the difficulty clinical practitioners face in interpreting unexplained predictive models, this study used the Shapley additive explanations (SHAP) method to elucidate the XGBoost model by calculating the contribution of each feature variable to the predicted outcome (Figure 5). The variables showing the contribution to delayed extubation after general anesthesia in the SHAP plot of the XGBoost model were as follows: age, BMI, ASA classification, tympanic temperature following PACU admission, sex, intraoperative infusion volume, duration of surgery, surgical site, surgical level, case condition, cerebral stroke history, sufentanil administered before extubation in the PACU, and history of COPD (Figure 6).

## Discussion

### Principal Findings

This study collected perioperative assessment and management data, including nursing management indicators in the PACU, and attempted to construct predictive models for delayed extubation in the PACU using various machine learning algorithms. The results showed that the XGBoost-based model achieved the highest predictive performance. The SHAP interpretation in the XGBoost model identified features with high predictive value, providing a basis for early risk assessment of delayed extubation and optimal nursing intervention strategies within the PACU.

The SHAP summary plot demonstrated that age, BMI, ASA classification, and tympanic temperature were the top 4 features in predicting delayed extubation risk. Age showed the strongest positive contribution to model predictions, indicating that patients aged ≥65 years were more likely to experience delayed extubation. This finding aligns with previous studies showing that older patients are at higher risk for delayed extubation due to decreased respiratory reserve, altered drug metabolism, and delayed muscle strength recovery [18,19]. PACU nurses should implement individualized monitoring and interventions for older adults or patients at risk of respiratory dysfunction (eg, obesity, obstructive sleep apnea, and COPD), including close monitoring of respiratory rate, oxygen saturation, and partial pressure of end-tidal carbon dioxide to detect hypoventilation, hypoxemia, or airway obstruction early, as well as extending awakening time to ensure full consciousness and respiratory recovery before extubation [20,21]. Diaphragm ultrasound can effectively assess diaphragmatic thickness, thickening fraction, and mobility, reflecting diaphragm contractility, and is a powerful tool to predict the recovery of spontaneous breathing and extubation success [22]. In the PACU, particularly for older patients or those with impaired respiratory function (eg, COPD or chronic respiratory failure), diaphragmatic function assessment helps nurses determine whether the patient has sufficient respiratory drive and muscle strength to maintain ventilation after extubation [23]. If the thickening fraction is <20% or diaphragmatic movement is significantly reduced, it indicates diaphragmatic dysfunction and suggests that the patient may not yet be ready for extubation [24]. Conversely, normal diaphragmatic movement indicates good respiratory muscle recovery and extubation safety, reducing reintubation risks.

BMI was the second most important predictor. SHAP interpretation showed that underweight (BMI <18.5 kg/m²) contributed more to delayed extubation predictions than obesity. This contrasts with most studies focusing on obesity as a risk factor for delayed extubation. Previous studies indicated that patients with obesity have impaired lung function, characterized by decreased lung compliance and reduced functional residual capacity, which is further exacerbated by general anesthesia and mechanical ventilation, leading to delayed recovery of spontaneous breathing and increased risk of extubation delay [25,26]. However, patients who are underweight may also experience delayed extubation due to potential mechanisms such as malnutrition-induced respiratory muscle weakness, reduced adipose tissue affecting the distribution volume and metabolism of lipophilic anesthetics, and poor overall recovery capacity [27]. Therefore, preoperative nutritional risk screening (eg, Nutritional Risk Screening 2002 or Patient Generated Subjective Global Assessment) should be routinely performed to identify potential malnutrition [28]. During PACU recovery, nurses should closely assess muscle strength recovery, including respiratory and limb muscle strength, to evaluate readiness for extubation. Patients with respiratory muscle weakness, hypoventilation, or delayed awakening may require prolonged mechanical ventilation support to ensure sufficient respiratory capacity and consciousness before extubation, thus ensuring safety and reducing postoperative complications. ASA classification also contributed significantly to the model, with patients classified as ASA III or above having a higher likelihood of delayed extubation, suggesting that baseline comorbidities and overall health status play crucial roles in postoperative recovery [29]. PACU nurses should integrate comprehensive preoperative functional assessments with ASA classification to develop graded nursing plans, optimize resource allocation, and strengthen cardiopulmonary monitoring and support [30]. Tympanic temperature was also an important predictor, with lower temperatures associated with increased risk of delayed extubation. Previous studies have shown that hypothermia reduces hepatic and renal blood flow, slowing anesthetic drug metabolism and clearance and prolonging awakening and spontaneous breathing recovery [18,31]. PACU nurses should measure tympanic temperature immediately upon admission and initiate active warming using forced-air warming blankets and prewarmed infusions if the temperature is <36 °C. Temperature should be remeasured every 15 minutes during anesthesia recovery to ensure a stable warming trend, and a standardized hypothermia management protocol should be implemented to ensure continuity of care [32].

Furthermore, intraoperative infusion volume ≥1135 ml, anesthesia duration ≥230.5 minutes, neurosurgery, and higher surgical levels contributed moderately to predictions, suggesting that intraoperative fluid management, anesthesia duration, and surgical complexity should be comprehensively considered to optimize extubation timing. Increased intraoperative fluid volume may lead to interstitial fluid retention, edema, and decreased lung compliance, adversely affecting respiratory function and delaying recovery of spontaneous breathing and readiness for extubation [33]. Previous studies have shown that excessive fluid management is associated with increased risk of postoperative pulmonary complications [34]; thus, rational intraoperative fluid management is essential to avoid volume overload.

Finally, although a history of cerebral infarction, additional sufentanil administration in the PACU, and COPD showed relatively low SHAP contributions, they remain clinically important predictors. A history of cerebral infarction indicates potential central nervous system dysfunction, including altered consciousness, impaired swallowing, and weakened cough reflex, increasing extubation risks [35,36]. Previous studies have shown that individuals who have sustained a stroke are prone to delayed extubation and insufficient airway protection due to intracranial pressure changes, impaired autonomic regulation, and reduced respiratory center control [37,38]. Additional sufentanil administration in the PACU reflects increased postoperative analgesic requirements. While sufentanil is a potent lipophilic μ-opioid receptor agonist providing excellent analgesia, high doses may suppress respiratory drive and delay spontaneous breathing recovery [39]. Therefore, postoperative analgesia should aim to achieve effective pain control while minimizing the risk of respiratory depression. For older patients or those with compromised respiratory function, sufentanil dosing should follow recommended ranges (0.1‐0.5 μg/kg), with close monitoring of respiratory status and consciousness to ensure effective analgesia without prolonging extubation time [40]. Patients with COPD are prone to airway secretion retention, ventilation-perfusion mismatch, and weakened respiratory effort due to abnormal airway function and increased resistance. They may experience respiratory muscle fatigue and hypoxemia after extubation, necessitating reintubation. PACU staff typically delay extubation until oxygenation improves, necessitating careful assessment of respiratory function recovery and extubation timing, thus increasing the risk of delayed extubation [41].

### Strengths and Limitations

This study found that among all models compared, XGBoost achieved the best performance. Previous models were mainly developed based on logistic regression and presented as nomograms [9]. However, XGBoost outperforms logistic regression in capturing complex nonlinear interactions among variables, resulting in higher predictive accuracy. Compared with other machine learning algorithms, XGBoost offers advantages such as superior performance with high-dimensional sparse data, built-in LASSO and ridge regularization, cross-validation to prevent overfitting and enhance generalizability, and native handling of missing data without prior imputation [29].

This study also had limitations. First, as a retrospective study, it was subject to unavoidable biases and lacked external validation. Additionally, some variables potentially affecting extubation time were not included, and only patients with ASA ≤ III were enrolled, limiting generalizability. Future multicenter prospective studies with larger samples are needed to develop and validate delayed extubation prediction models in the PACU. Furthermore, despite the obvious class imbalance in this study, no resampling techniques (eg, Synthetic Minority Over-sampling Technique) or class weighting strategies were used during model training. To reduce the risk of majority class bias, we incorporated a threshold adjustment mechanism in the evaluation stage by imposing dual constraints on sensitivity and specificity (eg, sensitivity ≥0.60 and specificity ≥0.60) to select clinically meaningful thresholds. Although this post hoc adjustment does not address imbalance during training, it partially mitigates the majority class overfitting. Future studies will incorporate integrated balancing strategies to further improve model robustness and generalizability.

### Conclusions

The machine learning–based predictive model for delayed extubation risk after general anesthesia has significant clinical implications. It allows for early identification of patients with high risk, enabling personalized management and timely interventions to reduce complications. PACU nurses can consider integrating the model into routine postoperative care, especially in PACU settings, to improve decision-making and optimize resource allocation.

## Supplementary material

10.2196/72602 Multimedia Appendix 1 Comparison of baseline characteristics between the training and test sets.

10.2196/72602 Multimedia Appendix 2 Variable coding and risk direction for Shapley additive explanations interpretation.

## Abbreviations

ASA: American Society of Anesthesiologists
AUROC: area under the receiver operating characteristic curve
COPD: chronic obstructive pulmonary disease
KNN: k-nearest neighbor
LASSO: least absolute shrinkage and selection operator
LightGBM: light gradient boosting machine
NPV: negative predictive value
PACU: postanesthesia care unit
PPV: positive predictive value
RF: random forest
ROC: receiver operating characteristic
SHAP: Shapley additive explanations
XGBoost: extreme gradient boosting

## References

[1] Cook TM, Scott S, Mihai R (2010). Litigation related to airway and respiratory complications of anaesthesia: an analysis of claims against the NHS in England 1995-2007. Anaesthesia.

[2] Cook TM, Woodall N, Frerk C, Fourth National Audit Project (2011). Major complications of airway management in the UK: results of the Fourth National Audit Project of the Royal College of Anaesthetists and the Difficult Airway Society. Part 1: anaesthesia. Br J Anaesth.

[3] Anastasian ZH, Kim M, Heyer EJ, Wang S, Berman MF (2016). Attending handoff is correlated with the decision to delay extubation after surgery. Anesth Analg.

[4] Thomas E, Martin F, Pollard B (2020). Delayed recovery of consciousness after general anaesthesia. BJA Educ.

[5] Kobayashi N, Wagatsuma T, Shiga T, Toyama H, Ejima Y, Yamauchi M (2020). Age-related changes in factors associated with delayed extubation after general anesthesia: a retrospective study. JA Clin Rep.

[6] Shimamoto Y, Sanuki M, Kurita S, Ueki M, Kuwahara Y, Matsumoto A (2022). Factors affecting prolonged time to extubation in patients given remimazolam. PLoS ONE.

[7] Maisat W, Siriratwarangkul S, Charoensri A, Wongkornrat W, Lapmahapaisan S (2020). Perioperative risk factors for delayed extubation after acute type A aortic dissection surgery. J Thorac Dis.

[8] Corley A, Bull T, Spooner AJ, Barnett AG, Fraser JF (2015). Direct extubation onto high-flow nasal cannulae post-cardiac surgery versus standard treatment in patients with a BMI ≥30: a randomised controlled trial. Intensive Care Med.

[9] Tong C, Miao Q, Zheng J, Wu J (2023). A novel nomogram for predicting the decision to delayed extubation after thoracoscopic lung cancer surgery. Ann Med.

[10] De la Garza Ramos R, Nakhla J, Nasser R (2017). Factors associated with prolonged ventilation and reintubation in adult spinal deformity surgery. J Clin Neurosci.

[11] Jing X, Zhu Z, Fan H (2023). Impact of delay extubation on the reintubation rate in patients after cervical spine surgery: a retrospective cohort study. J Orthop Surg Res.

[12] Martin ND, Codner P, Greene W, Brasel K, Michetti C, AAST Critical Care Committee (2020). Contemporary hemodynamic monitoring, fluid responsiveness, volume optimization, and endpoints of resuscitation: an AAST critical care committee clinical consensus. Trauma Surg Acute Care Open.

[13] Raksakietisak M, Keawsai T, Sirivanasandha B (2019). Factors related to delayed extubation in cervical spine surgery in an academic hospital: a retrospective study of 506 patients. Asian J Anesthesiol.

[14] Huang H, Wang J, Zhu Y (2023). Development of a machine-learning model for prediction of extubation failure in patients with difficult airways after general anesthesia of head, neck, and maxillofacial surgeries. J Clin Med.

[15] Zhao QY, Wang H, Luo JC (2021). Development and validation of a machine-learning model for prediction of extubation failure in intensive care units. Front Med (Lausanne).

[16] Vannucci A, Riordan IR, Prifti K (2021). Prolonged time to extubation after general anaesthesia is associated with early escalation of care: a retrospective observational study. Eur J Anaesthesiol.

[17] Huang Y, Pepe MS (2009). Biomarker evaluation and comparison using the controls as a reference population. Biostatistics.

[18] Hopf HW (2015). Perioperative temperature management: time for a new standard of care?. Anesthesiology.

[19] Klotz U (2009). Pharmacokinetics and drug metabolism in the elderly. Drug Metab Rev.

[20] Apfelbaum JL, Silverstein JH, Chung FF (2013). Practice guidelines for postanesthetic care: an updated report by the American Society of Anesthesiologists Task Force on Postanesthetic Care. Anesthesiology.

[21] American Society of Anesthesiologists Task Force on Perioperative Management of Patients With Obstructive Sleep Apnea (2014). Practice guidelines for the perioperative management of patients with obstructive sleep apnea: an updated report by the American Society of Anesthesiologists Task Force on Perioperative Management of patients with obstructive sleep apnea. Anesthesiology.

[22] Ferrari G, De Filippi G, Elia F, Panero F, Volpicelli G, Aprà F (2014). Diaphragm ultrasound as a new index of discontinuation from mechanical ventilation. Crit Ultrasound J.

[23] Llamas-Álvarez AM, Tenza-Lozano EM, Latour-Pérez J (2017). Diaphragm and lung ultrasound to predict weaning outcome: systematic review and meta-analysis. Chest.

[24] Kim WY, Suh HJ, Hong SB, Koh Y, Lim CM (2011). Diaphragm dysfunction assessed by ultrasonography: influence on weaning from mechanical ventilation. Crit Care Med.

[25] De Jong A, Chanques G, Jaber S (2017). Mechanical ventilation in obese ICU patients: from intubation to extubation. Crit Care.

[26] Robba C, Bonatti G, Battaglini D, Rocco PR, Pelosi P (2019). Mechanical ventilation in patients with acute ischaemic stroke: from pathophysiology to clinical practice. Crit Care.

[27] Xiao Y, Xu J, Zhao C, Pan G (2022). Risk factors of prolonged mechanical ventilation in patients undergoing redo valve surgery. Heart Surg Forum.

[28] Wischmeyer PE, Carli F, Evans DC (2018). American Society for Enhanced Recovery and Perioperative Quality Initiative joint consensus statement on nutrition screening and therapy within a surgical enhanced recovery pathway. Anesth Analg.

[29] Ahn JM, Kim J, Kim K (2023). Ensemble machine learning of gradient boosting (XGBoost, LightGBM, CatBoost) and attention-based CNN-LSTM for harmful algal blooms forecasting. Toxins (Basel).

[30] Ellinger E, Meybohm P, Röder D (2021). [Perioperative anesthesiologic management: risk assessment and preoperative improvement of patient conditions]. Anasthesiol Intensivmed Notfallmed Schmerzther.

[31] Pezawas T, Rajek A, Plöchl W (2007). Core and skin surface temperature course after normothermic and hypothermic cardiopulmonary bypass and its impact on extubation time. Eur J Anaesthesiol.

[32] Ji N, Wang J, Li X, Shang Y (2024). Strategies for perioperative hypothermia management: advances in warming techniques and clinical implications: a narrative review. BMC Surg.

[33] Mythen MG, Swart M, Acheson N (2012). Perioperative fluid management: consensus statement from the enhanced recovery partnership. Perioper Med (Lond).

[34] Casado D, López F, Martí R (2010). Perioperative fluid management and major respiratory complications in patients undergoing esophagectomy. Dis Esophagus.

[35] Hegland KW, Davenport PW, Brandimore AE, Singletary FF, Troche MS (2016). Rehabilitation of swallowing and cough functions following stroke: an expiratory muscle strength training trial. Arch Phys Med Rehabil.

[36] Barnett HM, Davis AP, Khot SP (2022). Stroke and breathing. Handb Clin Neurol.

[37] Patrizz A, El Hamamy A, Maniskas M (2023). Stroke-induced respiratory dysfunction is associated with cognitive decline. Stroke.

[38] Duarte E, Messaggi-Sartor M, Grau-Sánchez J (2020). Cerebral infarct site and affected vascular territory as factors in breathing weakness in patients with subacute stroke. J Rehabil Med.

[39] Dong CS, Zhang J, Lu Q (2017). Effect of Dexmedetomidine combined with sufentanil for post-thoracotomy intravenous analgesia: a randomized, controlled clinical study. BMC Anesthesiol.

[40] Liu L, Li B, Cao Q (2020). Effects of additional intraoperative administration of sufentanil on postoperative pain, stress and inflammatory responses in patients undergoing laparoscopic myomectomy: a double-blind, randomized, placebo-controlled trial. J Pain Res.

[41] Jian L, Sheng S, Min Y, Zhongxiang Y (2013). Risk factors for endotracheal re-intubation following coronary artery bypass grafting. J Cardiothorac Surg.

