# Supplementary material for: Predicting Delayed Extubation After General Anesthesia in Postanesthesia Care Unit Patients Using Machine Learning: Model Development Study
Source: JMIR Med Inform. 2025 Nov 11;13:e72602. doi: 10.2196/72602 (PMC12604829; doi:10.2196/72602)
Supplement: Multimedia Appendix 1 [file medinform-v13-e72602-s001.docx]

**Comparison of baseline characteristics between train and test sets**

| Property | Training dataset(n=3346) | Testing dataset(n=1433) | P-value |
| --- | --- | --- | --- |
| Age  ＜18 years  18-64 years  ≥65 years | 528(15.8%)  2183(65.2%)  635(19%) | 233(16.3%)  911(63.6%)  289(20.2%) | 0.519 |
| Sex,n(%) |  |  | 0.778 |
| Male | 1647(49.2%) | 699(48.8%) |  |
| Female | 1699(50.8%) | 734(51.2%) |  |
| BMI,n(%)  ＜18.5kg/m2  18.5-23.9kg/m2  24-27.9kg/m2  ≥28kg/m2 | 526(15.7%)  1444(43.2%)  1000(29.9%)  376(11.2%) | 226(15.8%)  603(42.1%)  421(29.5%)  183(12.8%) | 0.498 |
| Tympanic temperature following PACU admission,n(%) |  |  | 0.762 |
| ＜36℃ | 1136(34%) | 493(34.4%) |  |
| ≥36℃ | 2210(66%) | 940(65.6%) |  |
| History of cerebral infarction,n(%) |  |  | 0.239 |
| Yes | 64(1.9%) | 35(2.4%) |  |
| No | 3282(98.1%) | 1398(97.6%) |  |
| Smoking,n(%) |  |  | 0.795 |
| Yes | 439(13.1%) | 192(13.4%) |  |
| No | 2907(86.9%) | 1241(86.6%) |  |
| History of COPD,n(%) |  |  | 0.311 |
| Yes | 60(1.8%) | 32(2.2%) |  |
| No | 3286(98.2%) | 1401(97.8%) |  |
| chest X-ray findings,n(%) |  |  | 0.268 |
| Yes | 912(27.3%) | 413(28.8%) |  |
| No | 2434(72.7%) | 1020(71.2%) |  |
| Surgical Level,n(%) |  |  | 0.108 |
| Level 1 surgery | 33(1.0%) | 12(0.8%) |  |
| Level 2 surgery | 235(7.0%) | 95(6.6%) |  |
| Level 3 surgery | 1460(43.6%) | 668(46.7%) |  |
| Level 4 surgery | 1084(32.4%) | 469(32.7%) |  |
| Level 5 surgery | 534(16.0%) | 189(13.2%) |  |
| Case typing,n(%) |  |  | 0.191 |
| Usual | 1978(59.1%) | 863(60.2%) |  |
| Urgent | 202(6.0%) | 78(5.4%) |  |
| Difficult | 1130(33.8%) | 467(32.6%) |  |
| Critical | 36(1.1%) | 25(1.8%) |  |
| Surgical site,n(%) |  |  | 0.654 |
| Head and neck | 1358(40.6%) | 572(39.9%) |  |
| Chest and back | 369(11.0%) | 149(10.4%) |  |
| Abdomen | 1208(36.1%) | 515(35.9%) |  |
| Buttocks and perineum | 255(7.6%) | 118(8.2%) |  |
| Limbs | 156(4.7%) | 79(5.5%) |  |
| ASA classification,n(%) |  |  | 0.453 |
| Ⅰ | 209(6.2%) | 83(5.8%) |  |
| Ⅱ | 2626(78.5%) | 1112(77.6%) |  |
| Ⅲ | 511(15.3%) | 238(16.6%) |  |
| Intraoperative infusion volume,n(%)  ＜1135ml  ≥1135ml | 2685(80.2%)  661(19.8%) | 1168(81.5%)  265(18.5%) | 0.312 |
| Intraoperative blood loss (ml) |  |  | 0.269 |
| (Median ± IQR) | 10.0[5.0,20.0] | 10.0[5.0,20.0] |  |
| Duration of surgery,n(%)  ＜230.5min  ≥230.5min | 2701(80.7%)  645(19.3%) | 1155(80.6%)  278(19.4%) | 0.921 |
| Sufentanil administered before extubation in the PACU,n(%)  ＜5μg  5-10μg  ＞10μg | 2791(83.4%)  525(15.7%)  30(0.9%) | 1203(83.9%)  219(15.3%)  11(0.8%) | 0.845 |
| Preoperative K concentration  (mmol/L) | 4.00±0.358 | 4.01±0.358 | 0.308 |
| Preoperative Ca concentration  (mmol/L) | 2.27±0.18 | 2.27±0.17 | 0.453 |
| Preoperative Cr concentration  (μmol/L) | 67.835±37.733 | 68.25±45.014 | 0.742 |
| Preoperative RDW-CV,n(%)  ＜12.85%  ≥12.85% | 1192(35.6%)  2154(64.4%) | 534(37.3%)  899(62.7%) | 0.280 |
| Preoperative RDW-SD,n(%)  ＜49fL  ≥49fL | 3217(96.1%)  129(3.9%) | 1366(95.3%)  67(4.7%) | 0.190 |
| Preoperative Hb,n(%)  ≥120.0mmol/L  90.0-119.9mmol/L  60.0-89.9mmol/L  30.0-59.9mmol/L | 2437(72.9%)  771(23.0%)  135(4.0%)  3(0.1%) | 1072(74.8%)  313(21.8%)  46(3.1%)  2(0.1%) | 0.360 |
| Preoperative ALT(U/L) |  |  | 0.952 |
| (Median ± IQR) | 16.0[12.0,25.0] | 16.0[11.0,25.0] |  |
| Preoperative AST(U/L) |  |  | 0.449 |
| (Median ± IQR) | 20.0[16.0,26.0] | 20.0[16.0,27.0] |  |
| Cerebral stroke history,n（%） |  |  | 0.348 |
| Yes | 208(6.2%) | 79(5.5%) |  |
| No | 3138(93.8%) | 1354(94.5%) |  |
