# Supplementary material for: Predicting Delayed Extubation After General Anesthesia in Postanesthesia Care Unit Patients Using Machine Learning: Model Development Study
Source: JMIR Med Inform. 2025 Nov 11;13:e72602. doi: 10.2196/72602 (PMC12604829; doi:10.2196/72602)
Supplement: Multimedia Appendix 2 [file medinform-v13-e72602-s002.docx]

**Multimedia Appendix 2.** Variable coding and risk direction for SHAP interpretation

| Variable | Coding in analysis (levels) | Feature value mapped to “high” (red) in Figure 5 | Risk direction (SHAP-based interpretation on the test set) |
| --- | --- | --- | --- |
| AGE | 1 = <18 years;  2 = 18–64 years;  3 = ≥65 years | code 3 (≥65 years) | Predicted risk increases across age categories; the ≥65-year group shows the largest positive contribution, the 18–64-year group a smaller positive contribution, and the <18-year group the lowest. |
| BMI | 1 = <18.5;  2 = 18.5–23.9;  3 = 24–27.9;  4 = ≥28 kg/m² | code 4 (≥28 kg/m^2^) | Higher BMI categories are associated with higher predicted risk relative to lower BMI categories. |
| ASA classification | 1 = I;  2 = II;  3 = III | code 3 (III) | Higher ASA class is associated with higher predicted risk relative to lower classes. |
| Tympanic temperature on PACU admission | 1 = <36 °C;  2 = ≥36 °C | code 2 (≥36 °C) | Temperature <36 °C is associated with higher predicted risk; ≥36 °C is associated with lower predicted risk |
| Gender | 1 = Male;  2 = Female | code 2 (Female) | Male is associated with higher predicted risk compared with female. |
| Intraoperative infusion volume | 1 = <1135 mL;  2 = ≥1135 mL | code 2 (≥1135 mL) | Infusion volume ≥1135 mL is associated with higher predicted risk compared with <1135 mL. |
| Duration of surgery | 1 = <230.5 min;  2 = ≥230.5 min | code 2 (≥230.5 min) | Longer duration (≥230.5 min) is associated with higher predicted risk compared with shorter duration. |
| Surgical site | 1 = Head and neck (reference);  2 = Chest and back;  3 = Abdomen;  4 = Buttocks and perineum;  5 = Limbs | Higher codes 2–5 red;  code 1 blue | Associations are category-specific;interpret each site relative to the reference level (Head and neck). |
| Surgical level | 1 = Level 1 surgery;  2 = Level 2 surgery;  3 = Level 3 surgery;  4 = Level 4 surgery;  5 = Level 5 surgery | Higher codes (4–5 red; 1 blue) | Higher surgical level/grade is associated with higher predicted risk relative to lower levels. |
| Case condition | 1 = Usual;  2 = Urgent;  3 = Difficult;  4 = Critical | Higher codes 2–4 red;  code 1 blue | Increasing severity (Urgent/Difficult/Critical) is associated with higher predicted risk compared with Usual. |
| Cerebral stroke history | 1 = Yes;  2 = No | Code 2 (No) red;  code 1 (Yes) blue | Presence of a history of stroke (Yes) is associated with higher predicted risk compared with No |
| Sufentanil administered before extubation in the PACU | 1 = <5 µg;  2 = 5–10 µg;  3 = >10 µg | Code 3 (>10µg) red;  code 1 blue | In this cohort, higher dose categories show predominantly negative SHAP contributions, indicating lower predicted risk relative to <5 µg. |
| History of COPD | 1 = Yes;  2 = No | Code 2 (No) red;  code 1 (Yes) blue | Presence of COPD (Yes) is associated with higher predicted risk compared with No. |
